# Homogeneous Core/Shell NiMoO_4_@NiMoO_4_ and Activated Carbon for High Performance Asymmetric Supercapacitor

**DOI:** 10.3390/nano9071033

**Published:** 2019-07-19

**Authors:** Jia Yi Dong, Jin Cheng Xu, Kwun Nam Hui, Ye Yang, Shi Chen Su, Lin Li, Xi Tian Zhang, Kar Wei Ng, Shuang Peng Wang, Zi Kang Tang

**Affiliations:** 1 Institute of Applied Physics and Materials Engineering, University of Macau, Avenida da Universidade, Taipa 999078, Macao SAR, China; 2 Institute of Optoelectronic Material and Technology, South China Normal University, Guangzhou 510631, China; 3 Department of Physics, Harbin Normal University, Harbin 150000, China

**Keywords:** nickel molybdate, core-shell structure, supercapacitor, hydrothermal method

## Abstract

Here, we report the extraordinary electrochemical energy storage capability of NiMoO_4_@NiMoO_4_ homogeneous hierarchical nanosheet-on-nanowire arrays (SOWAs), synthesized on nickel substrate by a two-stage hydrothermal process. Comparatively speaking, the SOWAs electrode displays superior electrochemical performances over the pure NiMoO_4_ nanowire arrays. Such improvements can be ascribed to the characteristic homogeneous hierarchical structure, which not only effectively increases the active surface areas for fast charge transfer, but also reduces the electrode resistance significantly by eliminating the potential barrier at the nanowire/nanosheet junction, an issue usually seen in other reported heterogeneous architectures. We further evaluate the performances of the SOWAs by constructing an asymmetric hybrid supercapacitor (ASC) with the SOWAs and activated carbon (AC). The optimized ASC shows excellent electrochemical performances with 47.2 Wh/kg in energy density of 1.38 kW/kg at 0–1.2 V. Moreover, the specific capacity retention can be as high as 91.4% after 4000 cycles, illustrating the remarkable cycling stability of the NiMoO_4_@NiMoO_4_//AC ASC device. Our results show that this unique NiMoO_4_@NiMoO_4_ SOWA has great prospects for future energy storage applications.

## 1. Introduction

Supercapacitors (SCs) have shown great promise as the next-generation power devices because of their extraordinary properties, including high-power density, rapid charging, and prolonged cycling life. Electrochemical double layer capacitors and pseudocapacitors are the two major types of SCs with different storage mechanisms [1,2]. The electrochemical double layer capacitors store energy mainly via ion adsorptions at the interface between the electrodes and electrolyte. In comparison, pseudocapacitors have shown advantages in achieving higher energy density and specific capacitance, owing to the quick and reversible redox reactions at the surface of the electrodes [3]. The most widely known pseudocapacitors are fabricated with MnO_2_ and RuO_2_. However, the high resistance and dissolution of MnO_2_ [4,5] in aqueous electrolytes as well as the high cost and toxicity of RuO_2_ [6,7] hinder them from being used on a large scale. In this context, hybrid devices consisting of faradaic and capacitive electrodes arise as a potential candidate that can draw on the advantages of both types of supercapacitors. To fully exploit the strengths of the hybrid devices, the choice of materials and device structures have to be carefully optimized. In terms of material choice, binary metal oxides such as MnMoO_4_, NiMoO_4_, NiCo_2_O_4_, and CoMoO_4_ have been extensively investigated, owing to their multiple oxidation states as well as significantly better electrical conductivity compared with single-component oxides, rendering them ideal for high-performance charge storage devices [8,9]. Among these binary metal oxides, NiMoO_4_ has attracted intense research interest, due to the superior electrochemical activity of Ni [3,10]. Although Mo does not contribute to the faradaic redox reactions that play the most significant role in the overall capacitance/capacity of the device, its high electrical conductivity facilitates the efficient transfer of charges during the process. SCs with respectable specific capacity have been demonstrated with NiMoO_4_ based nanosheets [11] and nanobundles [12]. To further enhance the electrochemical performances of NiMoO_4_, a more efficient device architecture is highly desired.

Among various device structures, well-aligned nanoarrays of electrochemically active materials like hydroxides and metal oxides grown on conductive substrates have shown superior advantages in energy storage, especially for SCs [13,14]. Specifically, the nanoarray architecture can provide a large specific surface area for ion diffusion and electron transport, thus resulting in high specific capacity [15,16,17,18,19]. Additionally, growing the active materials directly on the conductive substrates can ensure that the former can adhere to the substrate firmly. This makes the active materials more mechanically stable during the charge/discharge process [20,21]. Three-dimensional (3D) structures have been introduced to further enhance the energy storing capability of nanoarrays by increasing the active area for redox reactions [22,23,24,25]. Heterogeneous nanosheet-on-nanowire arrays (SOWAs), a type of hierarchical nanoarrays constructed with different materials in the nanowire core and nanosheet shell, have been widely studied for realizing high-performing SCs [26,27,28].

Compared with the heterogenous architecture, homogeneous SOWAs, with their entire hierarchical architecture made of the same materials, show great promise in lowering the intrinsic resistance by eliminating the surface potential barrier at the core/shell junction [29,30]. In spite of the potential advantages, very few works have been reported on the rational design of homogeneous SOWAs for SCs. Therefore, the pioneering study of such architecture on NiMoO_4_ is highly desirable. In this paper, we report the fabrication and characterizations of novel homogeneous NiMoO_4_@NiMoO_4_ hierarchical SOWAs supercapacitors. The SOWAs, comprising NiMoO_4_ nanowires covered with NiMoO_4_ nanosheets, are realized on a Ni substrate by a two-stage hydrothermal treatment. Impressively, the SOWAs show over 30% enhancements in specific capacity and noticeably better electrochemical behaviors comparing with the electrodes formed by pure NiMoO_4_ nanowire arrays. We do believe that the significative reduction in series resistance of our homojunction material is a benefit for charge carrier transport along the interconnected nanowire network. Observing these extraordinary properties, we further utilized the homogeneous SOWAs structure to fabricate a asymmetric hybrid supercapacitor (ASC), which displays excellent cycling stability and electrochemical performances superior to other reported devices with heterogeneous hierarchical structures. These promising results fully demonstrate the potential for the NiMoO_4_@NiMoO_4_ SOWAs to be used in energy storage applications, which require low resistive loss, fast operations, and good mechanical stability.

## 2. Experimental Section

### 2.1. Materials Synthesis

Rectangular nickel conductive substrates (2.4 cm × 3 cm) were ultrasonically cleaned with 6 M HCl acid (99% pure, Alfa Aesar, Ward Hill, MA, USA), ethanol (99% pure, Alfa Aesar, Ward Hill, MA, USA), and deionized (DI) water, each for fifteen minutes sequentially. NiMoO_4_ nanowire arrays were grown on a Ni substrate, using a single-step hydrothermal treatment. The synthesis typically starts with adding an aqueous solution of 0.05 M Ni(NO_3_)_2_·6H_2_O (99% pure, Alfa Aesar, Ward Hill, MA, USA) and 0.05 M Na_2_MoO_4_ (99% pure, Alfa Aesar, Ward Hill, MA, USA) solution under constant magnetic stirring. Nanowires with the desired density and aspect ratio were found to grow when a 25% v/v (ethanol/DI water) solvent was added. Then, the Ni substrate was immersed into 40 mL mixture precursor in an autoclave and kept at 140 °C for 6 h. Subsequently, the substrate was removed from the solution and washed with DI water. Finally, the sample was annealed for 2 h at 400 °C in flowing argon afterwards. The sample obtained with this process is denoted as Sample I.

### 2.2. Preparation of SOWAs

To synthesize the aligned SOWAs, the nanowire arrays in Sample I were utilized as the backbone for the growth of nanosheets via a secondary hydrothermal reaction. Briefly, Sample I was loaded into an autoclave containing 40 mL mixture of 0.0029 M of Ni(NO_3_)_2_·6H_2_O and 0.0029 M of Na_2_MoO_4_. The reactants were then kept for 3 h at 140 °C, followed by washing and annealing in conditions similar to those used in Sample I. These SOWAs are denoted as Sample II.

### 2.3. Preparation of Nanowire Arrays with Same Mass as Sample II

To illustrate that the difference in electrochemical performance between Samples I and II is not due to the slight mass increase of NiMoO_4_ in the latter, we fabricated another nanowire array with the same nominal mass as Sample II. The process started with loading a Ni substrate into a solution that consisted of Ni(NO_3_)_2_·6H_2_O and Na_2_MoO_4_ equally at 0.0529 M in 40 mL precursor, then heated up for 9 h at 140 °C. The resulting nanowire arrays were then washed and annealed in conditions similar to those used in Sample I. The obtained sample was denoted as Sample III.

### 2.4. Preparation of an AC Electrode

Commercial activated carbon, acetylene black, and conducting graphite with mass fractions of 80 wt%, 7.5 wt%, and 7.5 wt%, respectively, were mixed to obtain a homogeneous black powder. 25 µL poly (tetrafluoroethylene) in 325 µL ethanol was added subsequently. The final paste was then fixed onto a Ni substrate under 10 MPa and dried for 12 h at 80 °C.

### 2.5. Materials Characterization

The morphology and crystal structure were characterized by field emission scanning electron microscopy (FESEM, Sigma, Zeiss, Oberkochen, Germany), X-ray diffraction (XRD, Smartlab, Rigaku, Tokyo, Japan), and transmission electron microscopy (TEM, Talos F200X, FEI, Hillsboro, OR, USA). The surface area was calculated using the Brunauer-Emmett-Teller (BET, 3Flex, Micromeritics, Norcross, GA, USA) method within a relative pressure (P/P_0_) range of 0.05–0.45.

### 2.6. Electrochemical Measurements

Electrochemical performances such as the cyclic voltammetry (CV) and galvanostatic charge–discharge profiles (GCD) were obtained with an electrochemical analyzer (CHI 760E, Shanghai Chenhua, Shanghai, China) at ambient temperature. The measurements were performed in a three-electrode electrochemical cell, which contained a 1 M KOH aqueous solution (electrolyte), a standard Hg/HgO (reference electrode), and Pt foil (counter electrode). We carried out the electrochemical impedance spectroscopy (EIS) measurements utilizing an alternating-current (AC) source which could generate a voltage of 10 mV amplitude, varying in a frequency ranging between 0.01 kHz and 100 kHz. Several CV cycles were performed as an activation step before the actual data collection. Samples I, II, and III were employed directly as the working electrodes. The effective working area of the electrode, i.e., the area of immersion inside the electrolyte, was fixed at 1 cm^2^. We estimated the mass loading to be approximately 1.2 mg/cm^2^, 1.4 mg/cm^2^, 1.4 mg/cm^2^, respectively. Specific capacity (C_SC_, mA h/g) and areal capacity (C_AC_, mA h/cm^2^) of the three electrodes were examined in a three-electrode system by Equations (1) and (2):

(1) $$C_{SC}=\frac{2I\times \int vdt}{mV}$$

(2) $$C_{AC}=\frac{2I\times \int vdt}{SV}$$

where *m* and *S* are the mass and area of the active electrodes, *I* is the discharge current, and *∫* v*dt* represents the area enclosed under the discharge curves. For the two-electrode ASC systems, Equations (3) and (4) were used to calculate the energy and power densities, respectively. The mass loading of AC was around 2.8 mg/cm^2^, according to Equation (5).

(3) $$E=\frac{I\times \int vdt}{m\times 3.6}$$

(4) $$P=\frac{E}{t}\times 3600$$

(5) $$\frac{m_{+}}{m_{-}}=\frac{C_{S-}\Delta V_{-}}{C_{S+}\Delta V_{+}}$$

## 3. Results and Discussion

**Figure 1:**
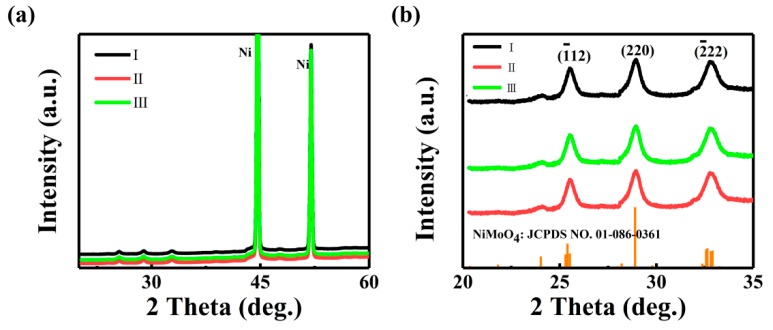
(**a**) X-ray diffraction (XRD) of Sample, Sample II, and Sample III grown on Ni foam; (**b**) Zoom-in view of XRD peaks from 20° to 35°.

The crystallographic phase of the three samples is first assessed using XRD (Figure 1a). Owing to the strong signal coming from the Ni substrate, the diffractions of the SOWAs cannot be readily resolved in Figure 1a. When zooming in to the regime between 20° and 35° (Figure 1b), one can clearly identify three diffractions which can be assigned to monoclinic NiMoO_4_. These diffractions are consistent with JCPDS data (Card No.86-0361) [31], exemplifying the purity of the NiMoO_4_ synthesized with the hydrothermal processes in all samples.

**Figure 2:**
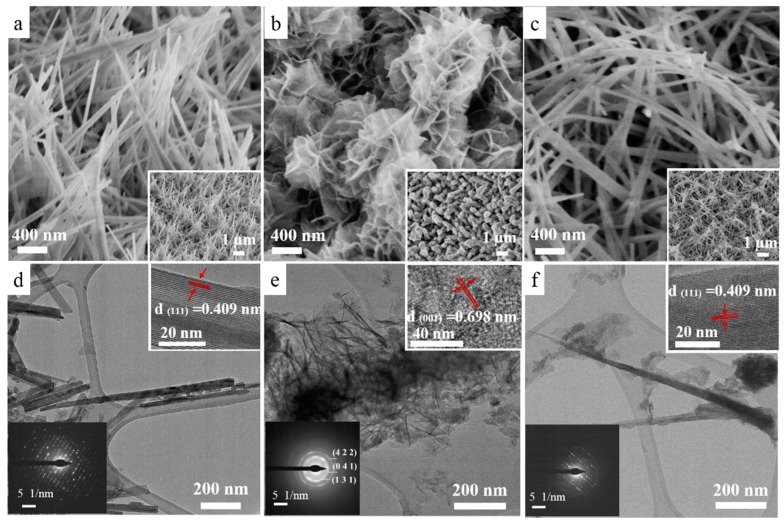
Field emission scanning electron microscopy (FESEM) images (**a**–**c**) of Samples I, II, and III. The insets show a large-area view of each sample. Transmission electron microscopy (TEM) images (**d**–**f**) of three samples. The insets in each TEM image show the corresponding selective area electron diffraction (SAED) pattern and high-resolution TEM (HRTEM) image.

It is well known that rod-like morphology can provide more reaction sites and improve ion transport [32,33,34]. To verify that our samples are indeed in the desired architecture, we extensively studied their morphology with SEM and TEM. Figure 2a,c shows representative SEM images revealing the morphologies of the three samples. From Figure 2a, we can see that NiMoO_4_ in Sample I grows into rods that generally align with the vertical direction and form an open network, which covers the entire substrate surface uniformly. Such nanowire framework provides a large area for redox reactions and charge storage. These capabilities are further enhanced by introducing the extra secondary NiMoO_4_ nano-flakes in Sample II. Impressively, as shown in Figure 2b, the NiMoO_4_ nanowire surface is completely covered with NiMoO_4_ nanosheet layers. These nanosheets significantly increase the density of active sites, thus enabling the full utilization of the active materials for energy storage. In Sample III, the extra mass of NiMoO_4_ for forming the 3D nanocomposite shell in Sample II is incorporated into the growth of nanowire arrays. As illustrated in Figure 2c, the nanowires become too long and lose the desired orthostatic morphology. The results here confirm that Sample II, an orthostatic nanowire network decorated with ultra-thin nanosheets, should present the ideal morphology for SC applications.

To get a clearer idea of the nanoscopic crystallinity of the hierarchical structures, we performed extensive TEM analysis. Figure 2d shows a typical 100-nm-thick NiMoO_4_ nanowire obtained from Sample I. The high-resolution TEM (HRTEM) image in the inset reveals clear lattice fringes exhibiting a periodicity of around 0.41 nm, which is in excellent agreement with the interplanar distance of the (111) planes of NiMoO_4_. The single-crystalline nature is further demonstrated with the selective area electron diffraction (SAED) pattern in Figure 2d. The excellent crystallinity is crucial for an efficient charge exchange and transport along the wires. Figure 2e demonstrates the architecture of Sample II comprising overlapping thin nanosheets, which agrees well with the SEM image in Figure 2b. Moreover, the SAED in Figure 2e exemplifies the polycrystallinity of Sample II with clearly indexable 422, 041, and 131 diffraction rings. The HRTEM image further confirms the short-term crystallinity of the nanosheets, which display lattice fringes separated by around 0.698 nm, which matches the (001) interplanar spacing NiMoO_4_. The lattice in Sample III (Figure 2f) is similar to those in Sample I, except that nanowires of Sample III are longer. Elemental mapping and line-scan by energy dispersive X-ray spectroscopy (Figure S1) of the central region of Sample II demonstrates that the nanostructures are chemically composed of Mo, O, and Ni, which are uniformly distributed over the entire sample. The TEM studies elucidate the crystalline properties of the NiMoO_4_ SOWAs, which are crucial to the electrochemical performances of the novel material system. The electron microscopy investigations imply that the SOWAs should possess the largest surface areas for charge exchange. We verify this by measuring the surface areas of the three samples quantitatively by BET measurement and the results are plotted in Figure S2. A BET surface area of 33.2 m^2^/g was observed for Sample II, which is over 20% larger than the surface areas of Sample I (26.2 m^2^/g) and Sample III (27.7 m^2^/g), as shown in the N_2_ adsorption–desorption profiles. The narrow hysteresis loops that appeared at different relative pressures are characteristics of hierarchical pores [35]. The relatively high specific surface area in Sample II not only increases the electrode/electrolyte contact area but also provides a lot more effective sites for redox reactions. These unique properties indicate that the SOWAs should exhibit superior electrochemical performance, which is to be discussed in more detail below.

**Figure 3:**
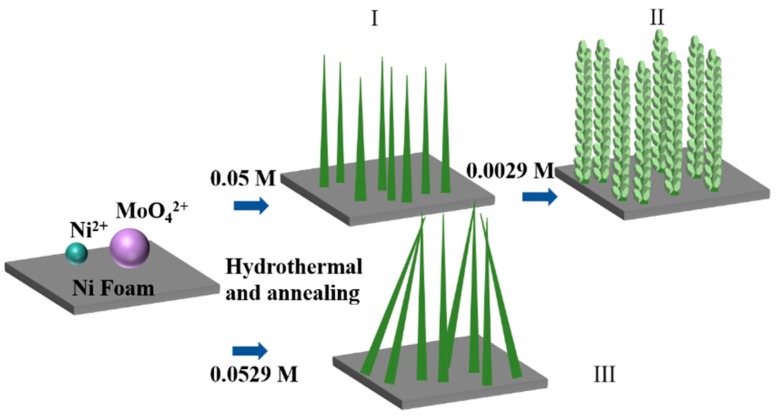
Illustration of the growth mechanism of Samples I, II, and III.

Based on the electron microscopy studies, we developed a growth mechanism as illustrated in Figure 3. First of all, a calcination process after a hydrothermal reaction gives rise to a densely packed nanowire array in Sample I. Subsequently, interconnected NiMoO_4_ nanosheets with various lateral sizes are grown on the NiMoO_4_ nanowire framework via a secondary hydrothermal treatment, resulting in the hierarchical structure in Sample II after a secondary calcination process. If all the reactants were supplied at the same time, the resulting nanowires would lose the desirable orthostatic property as in Sample III.

**Figure 4:**
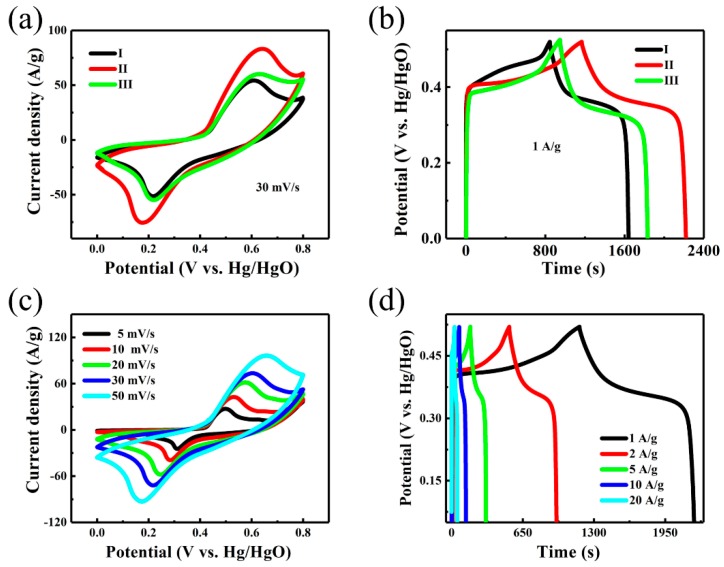
(**a**) Cyclic voltammetry (CV) profiles of Electrodes I, II, and III at 30 mV/s; (**b**) galvanostatic charge–discharge profiles (GCD) profiles of Electrodes I, II, and III at 1 A/g; (**c**) CV profiles of Electrode II at different sweep speeds; (**d**) GCD profiles of Electrode II at various current density.

Hydrothermal reaction time and the concentration of reactants undoubtedly play an important role in the morphology of the metal oxide electrodes, and thus affect their electrochemical performance. To validate the advantages of NiMoO_4_@NiMoO_4_ SOWAs, we investigated the electrochemical performances of the three samples. Electrodes I, II, and III were fabricated with Samples I, II, and III, respectively. Figure 4a displays the representative cyclic voltammetry (CV) profiles of the three electrodes within the voltage range of 0–0.8 V (vs. Hg/HgO) at 30 mV/s. Notably, Electrodes I and III show similar CV behaviors, indicating that the slightly larger mass in Electrode III does not have a big impact on the capacitance. In contrast, Electrode II shows more distinct redox peaks in its CV curve; besides, the obviously larger enclosed CV curve area in Electrode II than those in Electrodes I and III at identical sweep speed implies that the former has a much respectably higher specific capacitance. Indeed, the discharging time is –60% longer in Electrode II compared with the other two at 1 A/g in the GCD plot in Figure 4b. Although the CVs in Figure 4a were scanned within a voltage range of 0 to 0.8V, we set the voltage range for GCD at 0–0.52 V because considerable polarization is observed at voltage higher than 0.52 V [3,36]. To avoid such an undesirable effect, it is a usual practice to offset the voltage range of GCD from that used in the CV profiles [36,37,38,39].

The ultra-long discharge time elucidates the excellent electrochemical performance of Electrode II in accordance with Equations (1) and (2). To further investigate the electrochemical properties of Electrode II, we measured its CV at various sweep rates and GCD at various current levels. As represented in Figure 4c, all the CV profiles reveal meristic and clear redox peaks, which are strong indications that the specific capacitance characteristics predominantly originate from the faradic redox reactions of Ni^2+^/Ni^3+^ (Figure 4a,c) [40,41]. In addition, the redox peaks are prominent under low scanning rates (5 and 10 mV/s) but weaken at high sweep rates, due to the slow diffusion of OH^−1^ at the interface of electrolyte/electrode [42]. Hence, C_SC_ and C_AC_ obtained at the lowest scan rate can be considered as the closest to complete utilization of the electrode. Nevertheless, no significant change in the shape of the CV curves is observed when the scan rate increases and the peak current still increases proportionally, indicating that Electrode II can facilitate fast redox reactions. Additionally, the highly symmetric GCD profiles obtained at different current densities varying from 1 to 20 A/g (Figure 4d) clearly elucidate the excellent electrochemical behavior and reversible redox reaction activity.

**Figure 5:**
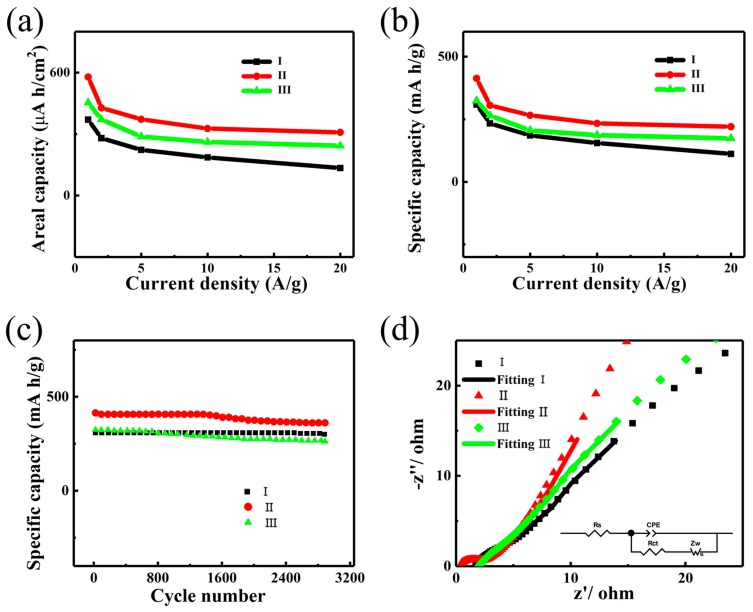
Electrochemical performances of the three Electrodes I, II, and III. Areal capacity (**a**) and specific capacity behavior (**b**) at various current density; (**c**) cycling behavior at 1 A/g; (**d**) the Nyquist plots and fitting curves in the frequency range of 0.01–100 kHz and the equivalent circuit diagram used for the analysis of electrochemical impedance spectroscopy (EIS) data.

Based on the above GCD behaviors, we calculate the areal capacity and specific capacity of the three electrodes and plot out their dependences on the current densities, as shown in Figure 5a,b. Since the effective contact area between the active materials and the electrolyte significantly diminishes at a high sweep rate, the capacity drops upon increasing current density [43,44,45]. The capacity of Electrode II can reach up to 413 mA h/g (578 µA h/cm^2^) at 1 A/g and drops to around 46.7% of the maximum value, i.e., 220 mA h/g (308 µA h/cm^2^) at 20 A/g. The maximum capacities of Electrodes I and III are much lower and can only reach 309 mA h/g (371 µA h/cm^2^) and 324 mA h/g (453 µA h/cm^2^), respectively, over 25% and 21% lower than that of Electrode II. Notably, the specific capacity of our homogeneous architecture here is favorably comparable with those of previously reported nanostructures, including CoMoO_4_@Co(OH)_2_ core-shell structures (265 mA h/cm^2^ at 2 mA/cm^2^) [46], as well as CoMoO_4_ nanoflakes (32.40 mA h/g; 492.48 µAh/cm^2^) [47], Ni–Mo–S nanosheets (312 mA h/g at 1 mA/cm^2^) [48], and flower-like Mn–Co oxysulfide (136 mA h/g at 2 A/g) [49]. Detailed comparisons in electrochemical performances among these devices and our structure can be found in Table S1. The superior energy storage performance of the NiMoO_4_@NiMoO_4_ SOWAs might be due to the larger specific surface area as well as more efficient charge carrier transport across the homojunction in our homogeneous hierarchical architecture.

The cycling stability of the three electrodes was assessed by GCD with the voltage ranging from 0 to 0.52 V, as displayed in Figure 5c. The capacity of Electrodes I, II, and III respectively drops by 1.5%, 4.3%, and 6.9% to 300.3 mA h/g, 361.2 mA h/g, and 262.3 mA h/g after 3000 cycles at a discharge current density of 1 A/g. Surprisingly, Electrode II experienced a relatively large cycling degradation, which could be attributed to the lack of stability in the structure of the NiMoO_4_ nanosheets upon high current operations. Nevertheless, the specific capacity of Electrode II stabilizes after around 3000 cycles and this value is still respectably higher than those of Electrodes I and III.

Aside from surface area, the impedance of the electrode also has an important effect on the overall behavior of SCs. The impedance properties of the electrodes were investigated in an open-circuit voltage with 5 mV amplitude, utilizing an EIS measurement [15,29,50,51]. Figure 5d displays the Nyquist plots measured in the frequency range of 0.01–100 kHz and the equivalent circuit is shown in the inset. Rs, CPE, Rct, and Zw correspond to the equivalent series resistance (ESR), constant phase element, charge-transfer resistance, and Warburg impedance, respectively. These elements basically define the various resistances that arose in the redox reactions. From the Nyquist plot, one can obtain the ESR from the *x*-intercept at the high frequency regime, which is a combined effect from the electrolyte resistance, the active material resistance, and the contact resistance between the electrode and electrolyte [50]. Apparently, Electrode II displays the smallest ESR, which is only 0.16 Ω. This very small ESR is more than 10 times lower than those of Electrode I (1.74 Ω) and Electrode III (2.03 Ω). It was also observed that the ESR of our homogeneous structure decreased by at least 68% compared with other heterogeneous structures or different morphologies of NiMoO_4_ (Table S1). Moreover, the diameter of the semicircle represents the interfacial charge-transfer resistance (Rct) [51]. In our case, the Rct of Electrodes I, II, and III are 3.0 Ω, 2.35 Ω, and 2.65 Ω, respectively, exemplifying that Electrode II has the best charge-transfer characteristics. Finally, the Nyquist plot of Electrode II exhibits a slope drastically steeper than those of Electrodes I and III at low frequencies. This validates the much better capacitive performance of Electrode II as a result of respectably lower diffusion resistance. These results suggest that the significantly better capability and stability of our homogeneous hierarchical structure can mainly be ascribed to the low resistances.

**Figure 6:**
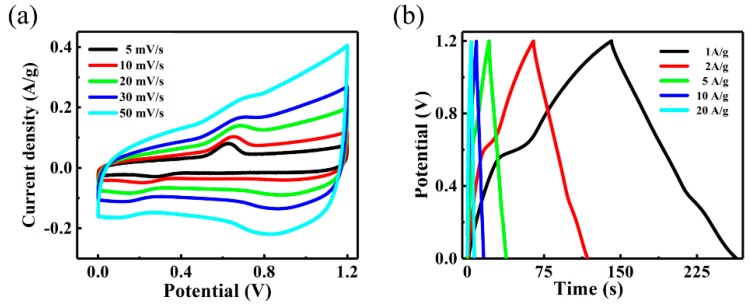
Electrochemical performance of NiMoO_4_@NiMoO_4_ nanosheet-on-nanowire arrays (SOWAs)//alternating-current (AC) asymmetric hybrid supercapacitor (ASC). (**a**) CV curves at various scan rates; (**b**) GCD curves at various current densities.

To assess the feasibility of the superior electrochemical properties of NiMoO_4_@NiMoO_4_ SOWAs for practical applications, we fabricated an asymmetric hybrid supercapacitor using the AC and optimized SOWAs as the negative and positive electrodes, respectively. The CV profiles of the ASC within 0 and 1.2 V at various sweep rates (5 to 50 mV/s) were displayed in Figure 6a. The CV profiles were tested at a constant scan rate of 30 mV/s within various ranges of voltage (see Figure S3). The peak tail, which appears as a consequence of an undesirable oxygen evolution reaction-induced peak in the CV profiles when the voltage is broadened to 1.5 V or higher, indicates that the maximum working voltage of the ASC should be around 1.2 V. Such a phenomenon was explained in a similar way in the previous literatures [52,53,54]. For example, Xu et al. reported that the optimized operating voltage range of the ZIF-LDH/GO//ZIF-C/G device was 0–1.6 V in order to avoid the oxygen evolution reaction at 1.6–1.8 V [55]. Elshahawy et al. reported that 1.6 V was utilized to be the upper limit of the operating voltage since, when the voltage went above 1.7 V, the oxygen evolution started [56]. It can be clearly seen that the overall capacitance of the NiMoO_4_@NiMoO_4_//AC ASC is composed of two components, namely the electrochemical double layer capacitors (EDLC)-type capacitance and Faradaic pseudocapacitance [50]. The shape of CV profiles show no distortion as the scan rate increases, indicating the desirable charging and discharging behaviors. The performance of GCD (Figure 6b) was also evaluated, while the voltage of ASC reached 1.2 V at a current density in between 2 and 40 A/g. The areal and specific capacity of the ASC are estimated on the basis of the discharge profiles, which are substantially higher than those ASCs reported before; for instance, ZnCo_2_O_4_@MnO_2_//α-Fe_2_O_3_ (0.40 F/cm^2^ at 2.5 mA/cm^2^), Co_0.85_Se//AC (0.33 F/cm^2^ at 1 mA/cm^2^), and NiCo_2_O_4_@Co_0.33_Ni_0.67_(OH)_2_//CMK-3 (0.89 F/cm^2^ at 5 mA/cm^2^) [57,58].

**Figure 7:**
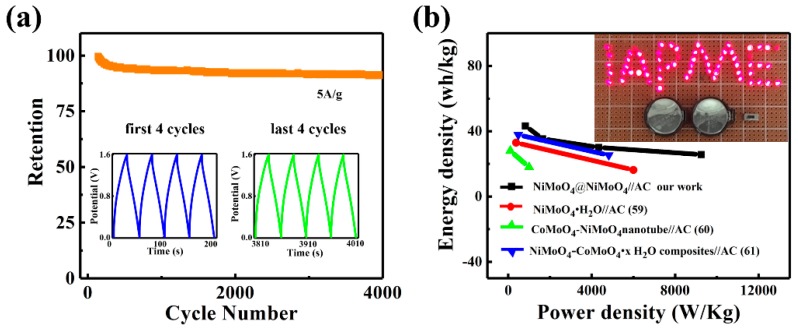
NiMoO_4_@NiMoO_4_//AC; (**a**) 4000 cycles cycling performance with the inset GCD plots of the first and last four circles; (**b**) the comparison of the Ragone plot with previous ASCs, and the inset picture shows two NiMoO_4_@NiMoO_4_//AC devices connected in series lighting up 64 red LEDs.

Cycling characteristics are another crucial factor that influences the performance of SCs. Figure 7a elucidates that the ASC device shows outstanding cycling stability. In particular, a 91.4% retention rate of the initial capacitance is obtained after 4000 cycles at a current density of 5 A/g. Furthermore, the repeated GCD profiles of the last four cycles show identical shape to the profiles of the first four cycles, as displayed in the inset of Figure 7a. The excellent capacitance stability of the ASC reveals that the NiMoO_4_@NiMoO_4_ SOWAs are suitable as stable electrode material. To obtain a more comprehensive assessment of electrochemical SCs, energy and power densities are also two important components that should be taken into account. Figure 7b shows the Ragone plot, which correlates the energy density with the power density of ASC devices. The energy density of Sample II, i.e., NiMoO_4_//AC ASC, can achieve 47.2 Wh/kg at 1.38 KW/kg, and still retains 25.7 Wh/kg at 9.25 KW/kg. Additionally, the energy density of our devices is compared with NiMoO_4_//AC ASC, as reported previously. As seen in Figure 7b, our devices show considerably higher energy density than NiMoO_4_·H_2_O//AC (17.72 Wh/kg) [59], CoMoO_4_-NiMoO_4_ nanotube//AC (33 Wh/kg at 375 W/kg) [60], and β-NiMoO_4_-CoMoO_4_·*x*H_2_O composites//AC (28 Wh/kg at 100 W/kg) [61]. The inset image of Figure 7b shows a demonstration of our ASC used in real operations. Two ASCs connected in series were encapsulated in a battery case and used to light up 64 red light emitting diodes connected in parallel for 137 s. The exciting results presented here fully elucidate the extraordinary electrochemical energy storage capability of the NiMoO_4_@NiMoO_4_//AC ASC.

## 4. Conclusions

In summary, uniform 3D NiMoO_4_@NiMoO_4_ SOWAs have been successfully demonstrated using a two-step hydrothermal and calcination process. The unique hierarchical architecture exhibits enhanced electrochemical behaviors, which can be ascribed to the increased reaction area and lower series resistance for carrier transport. In addition, the ASC device constructed with the SOWA demonstrates an impressive energy density of 47.2 Wh/kg at a power density of 1.38 kW/kg. These exciting results clearly indicate that the homogeneous SOWAs system can be used practically in constructing SCs with superior performance. In addition, the extraordinary synthesis tactics may give good guidance as to the construction of 3D nanostructures for implementing electrodes, which hold great promise for practical applications in electrochemical energy storage.

## Supplementary Materials

The following are available online at https://www.mdpi.com/2079-4991/9/7/1033/s1. Figure S1: High angle annular dark field (HAADF) TEM images of Sample II; Figure S2: N_2_ absorption-desorption isotherms of three samples; Figure S3: CV curves of NiMoO_4_@NiMoO_4_ SOWAs, AC electrode, and ASC device; Table S1: Comparison among our device with other heterostructure electrode materials.

## Abbreviations

NWSAs: nanowire/nanosheet arrays; ASC, asymmetric supercapacitor; AC, activated carbon; SCs, Supercapacitors; EDLCs, electrochemical double layer capacitors; FESEM, emission scanning electron microscopy; XRD, X-ray diffraction; BET, Brunauer-Emmett-Teller; CV, cyclic voltammetry; GCD, galvanostatic charge–discharge curves; EIS, Electrochemical impedance spectroscopy; SAED, area electron diffraction; HRTEM, high-resolution transmission electron microscopy.
