# Supplementary material for: Homogeneous Core/Shell NiMoO4@NiMoO4 and Activated Carbon for High Performance Asymmetric Supercapacitor
Source: Nanomaterials (Basel). 2019 Jul 19;9(7):1033. doi: 10.3390/nano9071033 (PMC6669477; doi:10.3390/nano9071033)
Supplement: Supplementary file 1 [file nanomaterials-09-01033-s001.pdf]

Supplementary Materials: Homogeneous Core/Shell $NiMoO_4$@$NiMoO_4$ and Activated Carbon for High Performance Asymmetric Supercapacitor

Jia Yi Dong [1], Jin Cheng Xu [1], Kwun Nam Hui [1], Ye Yang [1], Shi Chen Su [2], Lin Li [3], Xi Tian Zhang [3], Kar Wei Ng [1,*], Shuang Peng Wang [1,*] and Zi Kang Tang [1,*]

[1] Institute of Applied Physics and Materials Engineering, University of Macau, Avenida da Universidade, Taipa 999078, Macao SAR, China

[2] Institute of Optoelectronic Material and Technology, South China Normal University, Guangzhou 510631, China

[3] Department of Physics, Harbin Normal University, Harbin 150000, China

* Correspondence: billyng@um.edu.mo (K.W.N.); spwang@um.edu.mo (S.P.W.); zktang@um.edu.mo (Z.K.T.)

Content

Fig. S1 .... S-3
Fig. S2 .... S-4
Fig. S3 .... S-5
Table S1 .... S-**Error! Bookmark not defined.**

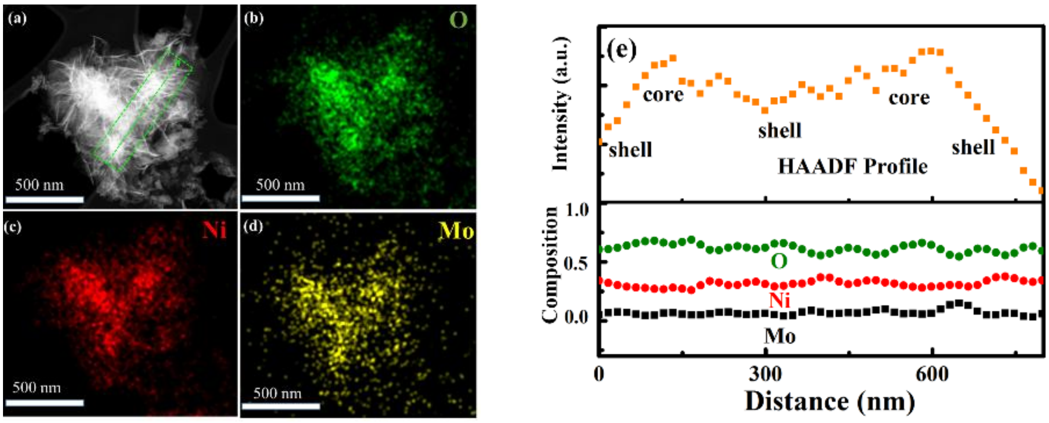

Figure S1. Images of the Sa**mple II**. High angle annular dark field (HAADF) TEM image (a) and (b-d) corresponding elemental mapping, revealing the homogeneous distribution of O, Ni and Mo in the sample. (e) Line-scan profiles obtained along the green dotted-line on core-shell $NiMoO_4$.

There is a significantly change in the intensity of HAADF when the green dotted-line was obtained along the sample. According to the mechanism of HAADF, the lighter areas with high intensity are core, and the dark areas in relatively low intensity positions are the shell, which means that the sample has an obvious core-shell structure. It also can be seen that despite the fluctuations due to thickness variations over the scan line, the components of elements in the core and shell were basically unchanged, indicating that the core-shell is composed of the same material. However, the content of nickel is higher than the value anticipated from the chemical formula due to the inevitable introduction of nickel from the Ni foam substrate during the preparation of TEM samples. Nevertheless, the exact composition of the material has been confirmed by XRD (Fig. 1) and thus we are confident that a homogeneous core/shell $NiMoO_4$ has been realized.

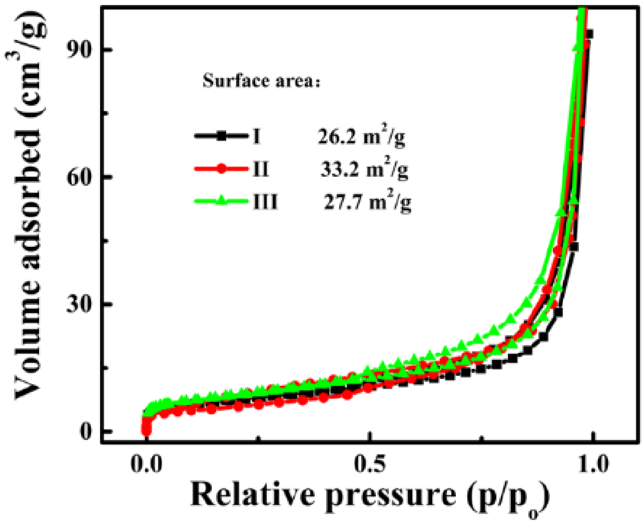

Figure S2. N_2 absorption-desorption isotherms of Samples **I, II, III.**

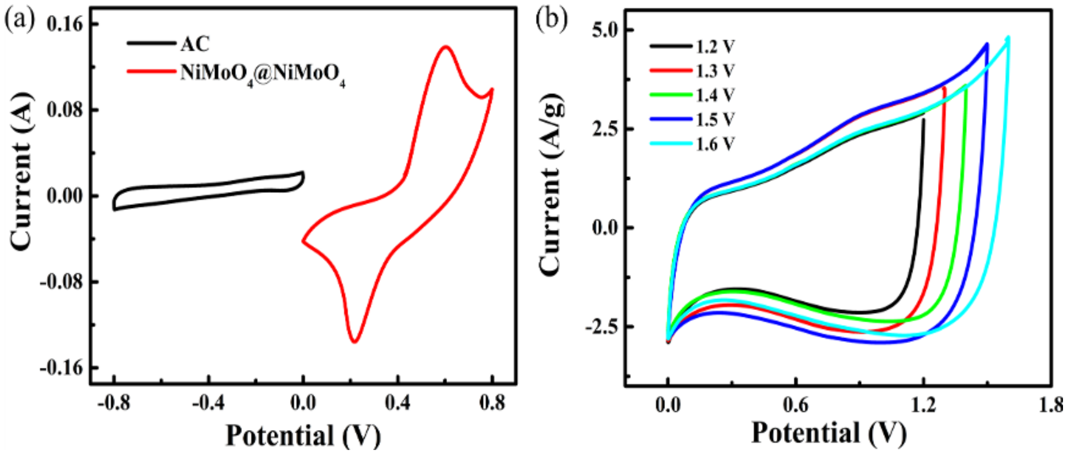

Figure S3. (a) CV curves of $NiMoO_4$@$NiMoO_4$ SOWAs and AC electrode with a scan rate of 30 mV/s; (b) CV curves of the ASC device collected at different scan voltage windows.

Table S1. Comparison among our device with other heterostructure electrode materials.

Electrode materials	Specific/areal capacity/capacitance	Cyclic stability	Energy density	Rs	Ref.
**$CoMoO_4$@$Co(OH)_2$ core-shell**	265 mA h/cm^2 at 2 mA/cm^2	98.64% retention after 2500 cycles	—	—	1
**$CoMoO_4$ Nanoflakes**	32.40 mA h/g (492.48 μAh/ cm^2)	85.98% retention after 5000 cycles	—	1.8 Ω	2
**Ni-Mo-S nanosheets**	312 mA h/g at 1 mA/cm^2	95.86% retention after 10000 cycles at 50 mA/cm^2	82.1 W h/ kg at 0.56 kW/kg	—	3
**Flower-like Mn-Co oxysulfide**	136 mA h/g at 2 A/g	86.5% retention after 3000 cycles at 20 A/g	—	—	4
**$NiMoO_4$@$Ni(OH)_2$ core/shell**	7.43 F/cm^2 at 4 mA/cm^2	72% retention after 1000 cycles at 8 mA/cm^2	—	0.5 Ω	5
**$NiMoO_4$ nanospheres**	974.4 F/g at 1 A/g	64% retention after 2000 cycles at 5 A/g	20.1 Wh/kg at 2100 W/kg	0.61 Ω	6
**β- $NiMoO_4$-$CoMoO_4$·xH_2O**	1472 F/g at a 5 mA/cm^2	92% retention after 1000 cycles at 10 mA/cm^2	28 Wh/kg at 100 W/kg	2.5 Ω	7
**$NiMoO_4$@$Co(OH)_2$ core/shell**	2.335 F/cm^2 at 5 mA/cm^2	83% retention after 5000 cycles at 20 mA/cm^2	—	2.0 Ω	8
**MnO_2@$NiMoO_4$ core-shell**	186.8 F/g at 10 mV/s	132.7% retention after 20000 cycles at 80 mV/s	32.5 Wh/kg at 0.75 KW/kg	1.5 Ω	9
**$MnCo_2O_4$@ MnO_2 core-shell**	858 F/g at 1 A/g	91% retention after 5000 cycles 2 A/g	6.0 Wh/kg at 252 W/kg	4.8 Ω	10
**$NiMoO_4$@ $NiMoO_4$ SOWAs**	413 mA h/g (578 μAh/cm^2) at 1 A/g	91.4 % retention after 4000 cycles at 5 A/g	47.2 Wh/kg at 1.38 kW/kg	0.16 Ω	Our work

References

1. G. K. Veerasubramani, A. Chandrasekhar, M. Sudhakaran, Y. S. Mok, S. J. Kim, *J. Mater. Chem. A* 2017, **5**, 11100.
2. M. Li, S. Xu, C. Cherry, Y. Zhu, D. Wu, C. Zhang, X. Zhang, R. Huang, R. Qi, L. Wang, *J. Mater. Chem. A*, 2015, **3**, 13776.
3. J. Balamurugan, C. Li, V. Aravindan, N. H. Kim, J. H. Lee, *Adv. Funct. Mater.*, 2018, **28**, 1803287.

4. M. Liu, Y. Fu, H. Ma, T. Wang, C. Guan, K. Hu, *Electrochim. Acta,* 2016, **191**, 916.
5. G. Jiang, M. Zhang, X. Li, H. Gao, *RSC Adv.*, 2015, **5**, 69365.
6. D. Cai, D. Wang, B. Liu, Y. Wang, Y. Liu, L. Wang, H. Li, H. Huang, Q. Li, T. Wang, *ACS applied materials & interfaces,* 2013, **5**, 12905.
7. Y. Qiao, J. Tu, X. Wang, D. Zhang, J. Xiang, Y. Mai, C. Gu, J. Power Sources, 2011, **196**, 7715.
8. W. Ren, D. Guo, M. Zhuo, B. Guan, D. Zhang, Q. Li, *RSC Adv.* 2015, **5**, 21881.
9. X. Wang, H. Xia, J. Gao, B. Shi, Y. Fang, M. Shao, *J. Mater. Chem. A* 2016, **4**, 18181.
10. X. Zheng, Y. Ye, Q.Yang, B. Geng, X. Zhang, *Dalton Trans.,* 2016, **45**, 572.
